# Cognitive Impairment and Age-Related Vision Disorders: Their Possible Relationship and the Evaluation of the Use of Aspirin and Statins in a 65 Years-and-Over Sardinian Population

**DOI:** 10.3389/fnagi.2014.00309

**Published:** 2014-11-07

**Authors:** Antonella Mandas, Rosa Maria Mereu, Olga Catte, Antonio Saba, Luca Serchisu, Diego Costaggiu, Enrico Peiretti, Giulia Caminiti, Michela Vinci, Maura Casu, Stefania Piludu, Maurizio Fossarello, Paolo Emilio Manconi, Sandra Dessí

**Affiliations:** ^1^Dipartimento di Scienze Mediche, Università degli Studi di Cagliari, Cagliari, Italy; ^2^Divisione Geriatria, Centro Alzheimer e disturbi della Memoria, P.O. SS. Trinità, Cagliari, Italy; ^3^Dipartimento di Scienze Chirurgiche e Odontoiatriche, Clinica Oculistica, Università degli Studi di Cagliari, Cagliari, Italy

**Keywords:** age-related vision disorders, dementia, statins, aspirin, elderly

## Abstract

Neurological disorders (Alzheimer’s disease, vascular and mixed dementia) and visual loss (cataract, age-related macular degeneration, glaucoma, and diabetic retinopathy) are among the most common conditions that afflict people of at least 65 years of age. An increasing body of evidence is emerging, which demonstrates that memory and vision impairment are closely, significantly, and positively linked and that statins and aspirin may lessen the risk of developing age-related visual and neurological problems. However, clinical studies have produced contradictory results. Thus, the intent of the present study was to reliably establish whether a relationship exist between various types of dementia and age-related vision disorders, and to establish whether statins and aspirin may or may not have beneficial effects on these two types of disorders. We found that participants with dementia and/or vision problems were more likely to be depressed and displayed worse functional ability in basic and instrumental activities of daily living than controls. Mini mental state examination scores were significantly lower in patients with vision disorders compared to subjects without vision disorders. A closer association with macular degeneration was found in subjects with Alzheimer’s disease than in subjects without dementia or with vascular dementia, mixed dementia, or other types of age-related vision disorders. When we considered the associations between different types of dementia and vision disorders and the use of statins and aspirin, we found a significant positive association between Alzheimer’s disease and statins on their own or in combination with aspirin, indicating that these two drugs do not appear to reduce the risk of Alzheimer’s disease or improve its clinical evolution and may, on the contrary, favor its development. No significant association in statin use alone, aspirin use alone, or the combination of these was found in subjects without vision disorders but with dementia, and, similarly, none in subjects with vision disorders but without dementia. Overall, these results confirm the general impression so far; namely, that macular degeneration may contribute to cognitive disorders (Alzheimer’s disease in particular). In addition, they also suggest that, while statin and aspirin use may undoubtedly have some protective effects, they do not appear to be magic pills against the development of cognitive impairment or vision disorders in the elderly.

## Introduction

Dementia and vision loss among the elderly are major health care problems. Dementia is a term used to describe a series of conditions that can affect a person’s ability to think, remember, understand, make judgments, communicate, and interact socially (MDGuidelines and Reed Group, 2009). Any person can develop dementia, but it is more common after the age of 65 years (Ritchie and Lovestone, 2002). The best-known and most common type of dementia is Alzheimer’s disease, which is characterized by the presence of neurofibrillary plaques and tangles in the brain. It can be caused by a variety of genetic, environmental, and other factors. Vascular dementia is the second most common form and occurs either due to a narrowing or a complete blockage of blood vessels in the brain, which deprive brain cells of nutrients and oxygen (Lee, 2011). Pure vascular dementia is relatively uncommon; Alzheimer’s disease, exacerbated by cerebrovascular lesions, is more common (mixed dementia). Mild cognitive impairment is a stage of cognitive decline between normal age-related memory loss and dementia. People with mild cognitive impairment have memory and reasoning problems that are evident to others, but which do not interfere with everyday life (Petersen, 2011). Macular degeneration, cataract, and glaucoma, as well as diabetic retinopathy, are the main causes of blindness in the elderly, affecting approximately one person in three, by the age of 65 years (NIH Med Plus, 2008; Pascolini and Mariotti, 2011; WHO, 2014). Over the last few years, clinical and epidemiological studies have been done suggesting that dementia and visual impairment – Alzheimer’s disease and age-related macular degeneration in particular – may share common pathogenetic mechanisms (Blanks et al., 1996; Sarks et al., 1999; Dentchev et al., 2003). Age-related vision impairment has been found to be closely associated with cognitive and behavioral manifestations (Clemons et al., 2006). Nevertheless, other studies found no significant relationship between cognitive impairment and vision loss in the elderly (Duron and Hano, 2008). More recently, evidence has also suggested a protective effect of statins, aspirin alone, or in combination against Alzheimer’s disease and macular degeneration (Williams et al., 2000; Jonker and Smit, 2003). This is in line with post-mortem studies of brain tissue pointing to an inflammatory process associated with the extracellular amyloid plaques characteristic of Alzheimer’s disease pathology (Hoozemans et al., 2006), as well as with the role of inflammation in macular degeneration pathogenesis. (Anderson et al., 2002). By contrast, it has also been reported that simvastatin (prescribed for high cholesterol) can lead to glaucoma, and that patients who take statins are 27% more likely to develop cataracts (Selak et al., 2014).

Aspirin is a photosensitizing drug that absorbs light energy and undergoes a photochemical reaction resulting in chemical modification of tissue, which can increase susceptibility to cataracts and macular degeneration (Skrzypczak-Jankun et al., 2005). Data from a recent study of ours showed that macular degeneration patients present changes in lipid metabolism similar to those seen in Alzheimer’s disease ones, i.e., accumulation of neutral lipids in the cytoplasm of peripheral blood mononuclear cells (Peiretti et al., 2014). We found no evidence that statins confer protection against vision disorders or dementia (Peiretti et al., 2014). In disagreement with earlier reports (Hall et al., 2001; Baghdasarian et al., 2004), we found a definite positive association between statin use and the presence of dementia and vision disorders (Peiretti et al., 2014). In view of these many discrepancies, we conducted an observational study with the aim of: firstly, quantifying the association between the incidence and progression of age-related vision disorders and cognitive impairment; secondly, evaluating the impact of statin and aspirin use on different types of age-related vision and cognitive disorders.

## Materials and Methods

### Participants

In order to examine the relationship between cognitive function, dementia, and visual impairment, in this study we analyzed data from 1,168 subjects 65 years or older admitted between 2006 and 2013 to the outpatients clinic of the geriatric care unit at the University of Cagliari and to the one at Santissima Trinità Hospital in Cagliari. All participants under examination underwent dementia and/or vision problem evaluation. Subjects found to be free from dementia or free from vision problems were utilized as controls 1 and 2, respectively. Control 3 was a group with neither vision problems nor dementia. Dementia and vision problems were further subdivided into categories: type of dementia: mild cognitive impairment, Alzheimer’s disease, mixed dementia, vascular dementia; type of vision problem: cataract, glaucoma, diabetic retinopathy, macular degeneration. All participants under examination underwent comprehensive geriatric assessment; instruments used for comprehensive geriatric assessment are described below. All participants gave informed consent. Possible/probable diagnosis of Alzheimer’s disease was made according to the criteria of the National Institute of Neurological and Communicative Disorders and Stroke-Alzheimer’s Disease and Related Disorders Association Work Group (NINCDS-ADRDA) (McKhann et al., 1984). Diagnosis of possible/probable vascular dementia was made according to the criteria of the National Institute of Neurological Disorders and Stroke, and of the Association Internationale pour la Recherche et l’Enseignement en Neurosciences Work Group (NINDS-AIREN) (Roman et al., 1993). Differential diagnosis between Alzheimer’s disease, vascular dementia, and mixed dementia was always supported by neuroimaging evidence (computed tomography scan and/or nuclear magnetic resonance). Subjects with symptomatic pre-dementia, but that preserved ability to function independently in daily life, were referred to as mild cognitive impairment.

All subjects were screened at the Eye clinic, including ocular examination by slit lamp, visual acuity tests with Snellen chart, and intraocular pressure measurement with Goldmann applanation. Fundus evaluation was done with indirect −20 diopter lens, in order to check the posterior pole as well as the mid peripheral retina. More specific exams included crystalline opacification grading after pupil dilation with tropicamide 1% by slit lamp in order to ascertain lens opacity or type-specific opacity (Chylack et al., 1993). Color fundus picture was taken in order to classify the presence of any sign of retinal disease. Patients who were considered as having any sign of macular degeneration in color fundus photography – following the macular degeneration grading system (Bird et al., 1995) – were more closely evaluated, in a masked fashion by two observers, in order to be investigated, when necessary, with fluorescein angiography. Detailed patient history was recorded, using a questionnaire focusing on individual medical history. The presence of cardiovascular diseases such as dyslipidemia, hypertension, and diabetes and the use of aspirin and statins were established by reading medical history and report analysis. To determine aspirin and statin use, we also checked the drug boxes that each patient brought along when undergoing the outpatient visit. Statin users were defined as ones having used any drug in the class of 3-hydroxy-3-methyl-glutaryl-CoA reductase inhibitors including simvastatin, fluvastatin, atorvastatin, lovastatin, pravastatin, and rosuvastatin for at least 2 years. Aspirin users were defined as ones routinely having used cardioaspirin (100/150 mg per tablet) at least once a day for 2 years.

### Comprehensive geriatric assessment

All subjects underwent comprehensive geriatric assessment, consisting of:

1. Activities of Daily Living index (Shah et al., 1989), which measures functional status by quantifying patient performance in 10 activities of daily life. These activities can be grouped according to self-care (feeding, grooming, bathing, dressing, bowel and bladder care, and toilet use) and mobility (ambulation, transfers, and stair-climbing). Five-point increments are used in scoring, with a maximum score of 100, indicating that a patient is fully independent in physical functioning, and a minimum score of zero, representing a totally dependent, bed-ridden state.
2. Instrumental Activities of Daily Living scale (Lawton and Brody, 1969), which assesses independence in eight activities that are more cognitively and physically demanding than activities of daily living, including managing finances, taking medications, using telephones, shopping, using transportation, preparing meals, doing housework, and washing. A score of 8 indicates total autonomy, and 0, total dependence.
3. Mini Mental State Examination, which assesses cognitive status (corrected for age and education), 30 correct-answer points indicating cognitive deficit absence, and zero, maximum cognitive deficit. Subjects with a mini mental state examination score <24 were considered cognitively impaired (Folstein et al., 1975; Measso et al., 1993).
4. Geriatric Depression Scale, which assesses depression symptoms. Of the 15 items, scores of 0–4 are considered normal; 5–8 indicate mild depression; 9–11 indicate moderate depression; and 12–15 indicate severe depression (Yesavage et al., 1983).
5. Cumulative Illness Rating Scale (Parmelee et al., 1995), which assesses comorbidity. The cumulative illness rating scale uses a five-point ordinal scale (scores 1–5) to estimate the severity of pathology in each of 14 items representing individual body systems, including cardiac, vascular, respiratory, endocrine/metabolic, gastrointestinal (upper), gastrointestinal (lower), hepatic, genitourinary, musculoskeletal, ophthalmologic/otolaryngologic, renal, neurologic, psychiatric, and hypertension. Each system is assigned a value ranging from 1 (no impairment) to 5 (extremely severe). A total cumulative illness rating scale score is obtained by summing the scores for all 14 items. The maximum impairment (MI) score represents maximum organ damage. The comorbidity index score, which reflects the number of concomitant diseases, is derived from the total number of categories in which moderate or severe levels (grades 3–5) of a chronic disease are identified (range 0–14). The severity index reflects the overall severity of diseases, and the average rating of 13 disease categories, excluding psychiatric behavioral problems (range 1–5).

### Statistical analysis

Quantitative variables were shown as mean ± SD. Comparisons between these variables were made by Student’s *t*-test or variance analysis (ANOVA). If a major effect was observed, a *post hoc* test (Bonferroni) was carried out to identify significant differences between categories. An odds ratio (OD) chi-square test was also performed to identify statistically significant associations. All statistical tests were made using Excel’s data analysis tool. A *P*-value of 0.05 was considered statistically significant.

## Results

The population included 1,168 subjects 65 years or older, admitted between 2006 and 2013 to the outpatients clinic of the geriatric care unit at the University of Cagliari and to the one at Santissima Trinità Hospital in Cagliari, of which 336 men (29%) and 832 women (71%), with a mean age of 78.4 years (±7.1) and an age range from 65 to 100 years.

We initially analyzed comprehensive geriatric assessment data in various types of dementia (Alzheimer’s disease, mixed dementia, vascular dementia) as well as in mild cognitive impairment. Considered together, patients with dementia (*n* = 551) had lower mini mental state examination scores, activities of daily living and instrumental activities of daily living scores, and higher geriatric depression scale scores than Control 1. The average age of patients and of Control 1 was not statistically different (data not shown). In mild cognitive impairment subjects, mini mental state examination scores and instrumental activities of daily living scores were significantly lower than those of Control 1, but higher than Alzheimer’s disease, mixed dementia, and vascular dementia patients’ (Table 1). Interestingly, Alzheimer’s disease patients differ significantly from vascular dementia patients regarding the geriatric depression scale. Similarly, regarding the cumulative illness rating scale and severity and comorbidity indices, Alzheimer’s disease patients’ scores were lower than vascular dementia and mixed dementia patients’, indicating that Alzheimer’s disease patients are less depressed, have a lower comorbidity level, both regarding global severity and the number of comorbidities (Table 1). As expected, subjects with any type of age-related vision disorders (*n* = 438), cataract, macular degeneration, glaucoma, and diabetic retinopathy, were more likely to be depressed, but, surprisingly, had significantly lower mini mental state examination scores than Control 2 (data not shown). Comparison of comprehensive geriatric assessment parameters by ANOVA regarding the various types of vision disorders showed significant differences in mini mental state examination, geriatric depression scale, instrumental activities of daily living, and comorbidity index scores. The *post hoc* Bonferroni test did not reach statistical significance for any of the estimated parameters (Table 2). However, when a *t*-test was performed, mini mental state examination scores were statistically significantly lower (*P* = 0.008) in macular degeneration patients than Control 2, and cataract and retinopathy patients’ geriatric depression scale scores were significantly higher (*P* = 0.005 and 0.042, respectively) than Control 2.

**Table 1:** **Comprehensive geriatric assessment testing for subjects with different types of dementia**.

	1	2	3	4	5	ANOVA *P*-value	Bonferroni at 0.05
	Controls 1 (*n* = 436)	MCI (*n* = 181)	AD (*n* = 230)	MD (*n* = 126)	VD (*n* = 195)		
Age (years)	78.8 ± 7.5	77.7 ± 6.8	78.5 ± 7.0	77.8 ± 6.4	78.4 ± 6.5	ns	ns
MMSE score	27.4 ± 1.7	24.0 ± 2.6	17.0 ± 5.4	17.3 ± 4.5	18.0 ± 4.8	**0.000**	1 vs. 2, 3, 4, 5
							2 vs. 3, 4, 5
GDS score	8.2 ± 3.9	8.8 ± 3.9	8.0 ± 4.4	8.6 ± 4.5	9.7 ± 3.5	**0.000**	1 vs. 5
							3 vs. 5
ADLs score	71.2 ± 22.9	68.5 ± 20.9	67.2 ± 24.6	67.2 ± 24.2	62.9 ± 24.1	**0.001**	1 vs. 5
IADL score	3.6 ± 2.4	2.7 ± 2.0	2.0 ± 1.9	1.9 ± 1.9	1.9 ± 1.7	**0.000**	1 vs. 2, 3, 4, 5
							2 vs. 4, 5
CIRS score	31.0 ± 4.7	32.1 ± 4.6	28.8 ± 6.7	31.5 ± 5.5	32.4 ± 4.3	**0.000**	1 vs. 5
							3 vs. 1, 2, 4, 5
CIRS-MI	3.6 ± 0.5	3.6 ± 0.5	3.6 ± 0.5	3.7 ± 0.5	3.7 ± 0.5	ns	ns
CIRS-SI	2.2 ± 0.3	2.2 ± 0.4	2.0 ± 0.5	2.2 ± 0.4	2.3 ± 0.3	**0.000**	3 vs. 1, 2, 4, 5
CIRS-CI	6.7 ± 2.1	7.2 ± 2.2	5.6 ± 2.9	6.9 ± 2.5	7.3 ± 2.0	**0.000**	1 vs. 3, 5
							3 vs. 2, 4, 5

*Results are shown as mean ± SD*.

*Statistically significant differences according to ANOVA are indicated in bold type*.

*MCI, mild cognitive impairment; AD, Alzheimer’s disease; MD, mixed dementia; VD, vascular dementia; MMSE, mini mental state examination; GDS, geriatric depression scale; ADL, activities of daily living; IADL, instrumental activities of daily living; CIRS, cumulative illness rating scale; CIRS-MI, maximum impairment; CIRS-SI, severity index; CIRS-CI, comorbidity index*.

**Table 2:** **Comprehensive geriatric assessment parameters in subjects with different types of age-related vision disorders**.

	1	2	3	4	5	ANOVA *P*-value	Bonferroni at 0.05
	Control 2 (*n* = 730)	Cataract (*n* = 195)	AMD (*n* = 119)	Glaucoma (*n* = 51)	Retinopathy (*n* = 73)	
Age (years)	78.5 ± 6.7	78.3 ± 8.4	78.4 ± 6.9	77.9 ± 8.2	78.2 ± 6.7	ns	ns
MMSE score	22.5 ± 6.0	22.0 ± 5.5	20.9 ± 6.3	21.3 ± 5.9	21.5 ± 5.9	**0.044**	ns
GDS score	8.3 ± 4.0	9.2 ± 4.0	8.6 ± 3.9	8.3 ± 4.0	9.3 ± 4.0	**0.029**	ns
ADLs score	68.5 ± 24.4	65.7 ± 21.5	68.5 ± 21.9	68.7 ± 23.1	70.1 ± 22.9	ns	ns
IADL score	2.8 ± 2.3	2.2 ± 1.9	2.4 ± 2.0	3.0 ± 2.1	2.4 ± 2.0	**0.004**	ns
CIRS score	30.6 ± 5.1	32.2 ± 4.7	30.6 : 6.2	32.6 ± 5.3	32.0 ± 6.4	ns	ns
CIRS-MI	3.6 ± 0.5	3.7 ± 0.5	3.6 ± 0.5	3.6 ± 0.5	3.5 ± 0.5	ns	ns
CIRS-SI	2.1 ± 0.4	2.2 ± 0.3	2.1 ± 0.5	2.3 ± 0.4	2.2 ± 0.5	ns	ns
CIRS-CI	6.5 ± 2.3	7.0 ± 2.3	6.5 ± 2.9	7.6 ± 2.2	7.2 ± 3.1	**0.000**	ns

*Results are shown as mean ± SD*.

*Statistically significant differences according to ANOVA are indicated in bold type*.

*AMD, age-related macular degeneration; MMSE, mini mental state examination; GDS, geriatric depression scale; ADL, activities of daily living; IADL, instrumental activities of daily living; CIRS, cumulative illness rating scale; CIRS-MI, maximum impairment; CIRS-SI, severity index; CIRS-CI, comorbidity index*.

Considering that a large number of studies have suggested that subjects with cardiovascular diseases have a higher risk of dementia than those without cardiovascular disease, we also tested to see whether a relationship existed between the various types of dementia (Table 3) and dyslipidemia, general atherosclerosis, diabetes, and hypertension. Compared with Control 1, none of these individual diseases were positively associated with any type of dementia or with Alzheimer’s disease. Significant association was found between atherosclerosis and mild cognitive impairment, mixed dementia, and vascular dementia, and one between diabetes and mixed dementia (Table 3).

**Table 3:** **Relationship between cardiovascular diseases and dementia**.

Participants	Total (*n* = 1168)	Control 1 (*n* = 436)	All types of dementia (*n* = 551)	MCI (*n* = 181)	AD (*n* = 230)	DM (*n* = 126)	DV (*n* = 195)
Dyslipidemia	No	*n* = 241	*n* = 195	*n* = 96	*n* = 123	*n* = 66	*n* = 109
	Yes	*n* = 394	*n* = 338	*n* = 85	*n* = 107	*n* = 60	*n* = 86
χ^2^			0.255	0.307	0.231	0.399	0.025
*P*-value			0.6137	0.5795	0.6310	0.5275	0.8743
OD (95%CI)			1.1 (0.8–1.3)	1.1 (0.8–1.5)	1.1 (0.8–1.5)	1.1 (0.8–1.7)	1.0 (0.7–1.4)
Atherosclerosis	No	*n* = 208	*n* = 228	*n* = 64	*n* = 121	*n* = 47	*n* = 76
	Yes	*n* = 308	*n* = 424	*n* = 117	*n* = 109	*n* = 79	*n* = 119
χ^2^			3.864	**9.411**	1.702	**5.158**	**4.920**
*P*-value			0.0493	**0.0022**	0.1920	**0.0231**	**0.0265**
OD (95% CI)			1.3 (1.0–1.6)	**1.7 (1.2–2.4)**	0.8 (0.6–1.1)	**1.5 (1.0–2.3)**	**1.4 (1.0–2.0)**
Diabetes	No	*n* = 311	*n* = 125	*n* = 127	*n* = 158	*n* = 74	*n* = 145
	Yes	*n* = 504	*n* = 228	*n* = 54	*n* = 72	*n* = 52	*n* = 50
χ^2^			0.875	0.100	0.590	**8.690**	0.731
*P*-value			0.3495	0.7516	0.4425	**0.0032**	0.3926
OD (95% CI)			1.1 (0.9–1.5)	1.1 (0.7–1.5)	1.1 (0.8–1.6)	**1.7 (1.2–2.6)**	0.9 (0.6–1.3)
Hypertension	No	*n* = 106	*n* = 330	*n* = 37	*n* = 65	*n* = 24	*n* = 41
	Yes	*n* = 167	*n* = 565	*n* = 144	*n* = 165	*n* = 102	*n* = 154
χ^2^			0.77	1.280	1.447	1.841	0.965
*P*-value			0.5395	0.2579	0.2291	0.1749	0.3259
OD (95% CI)			1.1 (0.8–1.4)	1.2 (0.8–1.9)	0.8 (0.6–1.2)	1.4 (0.8–2.2)	1.2 (0.8–1.8)

*Significant differences are in bold type*.

*MCI, mild cognitive impairment; AD, Alzheimer’s disease; MD, mixed dementia; VD, vascular dementia*.

As shown in Table 4, all types of dementia considered by us were found, taken collectively, to be positively associated with all types of vision disorders considered by us, taken collectively. Furthermore, Alzheimer’s disease, mixed dementia, and vascular dementia were found to be individually associated with all types of vision disorders considered by us, taken collectively. In addition, Alzheimer’s disease was found to be positively associated with macular degeneration; mixed dementia, with glaucoma and macular degeneration; and vascular dementia, with retinopathy. It is important to underline the association between Alzheimer’s disease and macular degeneration.

**Table 4:** **Associations between dementia and vision disorders**.

Participants	Total (*n* = 1168)	Control 1 (*n* = 436)	All types of dementia (*n* = 732)	MCI (*n* = 181)	AD (*n* = 230)	MD (*n* = 126)	VD (*n* = 195)
All vision disorders	No	*n* = 297	*n* = 433	*n* = 117	*n* = 135	*n* = 68	*n* = 113
	yes	*n* = 139	*n* = 299	*n* = 64	*n* = 95	*n* = 58	*n* = 82
χ^2^			**9.37**	0.70	**5.87**	**8.60**	**6.12**
*P-*value			**0.002**	0.40	**0.015**	**0.003**	**0.013**
OD (95% CI)			**1.5 (1.1–1.9)**	1.2 (0.8–1.7)	**1.5 (1.1–2.1)**	**1.8 (1.2–2.7)**	**1.5 (1.1–2.2)**
Cataract	No	*n* = 297	*n* = 433	*n* = 117	*n* = 135	*n* = 68	*n* = 113
	yes	*n* = 72	*n* = 123	*n* = 30	*n* = 32	*n* = 23	*n* = 38
χ^2^			0.90	0.053	0.009	1.838	2.053
*P-*value			0.340	0.817	0.924	0.175	0.151
OD (95% CI)			1.2 (0.8–1.6)	1.1 (0.7–1.7)	1.0 (0.6–1.5)	0.7 (0.4–12.2)	1.4 (0.9–2.2)
Glaucoma	No	*n* = 297	*n* = 433	*n* = 117	*n* = 135	*n* = 68	*n* = 113
	yes	*n* = 17	*n* = 34	*n* = 7	*n* = 5	*n* = 15	*n* = 7
χ^2^			1.03	0.007	0.73	**14.05**	0.024
*P-*value			0.381	0.935	0.391	**0.0002**	0.875
OD (95% CI)			1.4 (0.7–25)	1.0 (0.4–2.6)	1.6 (0.2–1.8)	**3.8 (1.8–8.0)**	1.1 (0.4–2.7)
Retinopathy	No	*n* = 297	*n* = 433	*n* = 117	*n* = 135	*n* = 68	*n* = 113
	yes	*n* = 19	*n* = 54	*n* = 15	*n* = 16	*n* = 6	*n* = 17
χ^2^			**5.97**	3.801	3.09	0.44	**6.19**
*P-*value			**0.014**	0.051	0.078	0.507	**0.013**
OD (95% CI)			**2.0 (1.1–3.4)**	2.1 (1.0–4.1)	1.8 (0.9–3.7)	1.4 (0.5–3.6)	**2.3 (1.2–4.7)**
AMD	No	*n* = 297	*n* = 433	*n* = 117	*n* = 135	*n* = 68	*n* = 113
	yes	*n* = 31	*n* = 88	*n* = 12	*n* = 42	*n* = 14	*n* = 20
χ^2^			**9.24**	0.002	**18.95**	**3.90**	3
*P-*value			**0.002**	0.960	**0.000**	**0.048**	0.083
OD (95% CI)			**1.9 (1.3–3.0)**	1.0 (0.5–2.0)	**3.0 (1.8–4.9)**	**2.0 (1.0–3.9)**	1.7 (0.9–3.1)
Statin users	No	*n* = 288	*n* = 401	*n* = 92	*n* = 124	*n* = 72	*n* = 113
	Yes	*n* = 148	*n* = 331	*n* = 89	*n* = 106	*n* = 54	*n* = 82
χ^2^			**14.35**	**12.53**	**9.40**	9.40	3.82
*P-*value			**0.0002**	**0.0004**	**0.002**	0.066	0.050
OD (95% CI)			**1.6 (1.3–2.0)**	**1.9 (1.3–2.7)**	**1.7 (1.2–2.3)**	1.5 (1.0–2.2)	1.4 (1.0–2.0)
Aspirin users	No	*n* = 259	*n* = 352	*n* = 85	*n* = 130	*n* = 47	*n* = 90
	Yes	*n* = 177	*n* = 380	*n* = 96	*n* = 100	*n* = 79	*n* = 105
χ^2^			**14.02**	**8.02**	0.51	**19.25**	**9.57**
*P-*value			**0.0002**	**0.005**	0.473	**0.000**	**0.002**
OD (95% CI)			**1.6 (1.2–2.0)**	**1.6 (1.2–2.3)**	1.1 (0.8–1.6)	**2.5 (1.6–3.7)**	**1.7 (1.2–2.4)**
Statin + Aspirin users	No	*n* = 192	*n* = 214	*n* = 35	*n* = 79	*n* = 37	*n* = 63
	yes	*n* = 81	*n* = 193	*n* = 40	*n* = 55	*n* = 43	*n* = 55
χ^2^			**21.39**	**14.52**	**5.22**	**15.74**	**10.42**
*P-*value			**0.000**	**0.0001**	**0.022**	**0.001**	**0.0012**
OD (95% CI)			**2.1 (1.5–3.0)**	**2.7 (1.6–4.6)**	**1.6 (1.1–2.5)**	**2.7 (1.6–4.6)**	**2.1 (1.3–3.2)**

*Significant differences are in bold types*.

*AMD, age-related macular degeneration; MCI, mild cognitive impairment; AD, Alzheimer’s disease; MD, mixed dementia; VD, vascular dementia*.

We considered the various associations by determining chi-square test. The associations between the dementia categories and aspirin use alone, statin use alone, and the combination of these last two, were found to be statistically positively significant compared to aspirin non-use. Similarly, aspirin use alone was found to be significantly associated with mild cognitive impairment, mixed dementia, and vascular dementia in particular. Compared to statin non-use, statin use alone was found to be significantly positively associated with all types of dementia considered by us, taken collectively, and also with Alzheimer’s disease. Furthermore, the combination of aspirin and statin use was associated with all types of dementia considered by us, taken collectively, and with mild cognitive impairment, Alzheimer’s disease, mixed dementia and vascular dementia in particular (Table 4).

The effects of aspirin use alone, statin use alone, and the combination of these were also assessed in patients with vision disorders but without any type of dementia (*n* = 150) and in Control 3 (*n* = 295). The number of patients with vision disorders taking aspirin alone, statin alone, and the combination of these was 55, 46, and 21, respectively; while the number of Control 3 taking aspirin alone, statin alone, and the combination of these was 118, 101, and 57, respectively.

Comparing Control 3 with patients with vision disorders but without any type of dementia, no significant association was found for statin use alone (chi-square test 0.573, *P* = 0.4490, OR = 1.18, 95% CI 0.77–1.80), for aspirin use alone (chi-square test 0.465, *P* = 0.4953, OR = 1.15, 95% CI 0.77–1.73), or for the combination of these (chi-square test 1.948, *P* = 0.1628, OR = 1.47, 95% CI 0.85–2.54).

Commonly prescribed statins are identified by their lipophilic or hydrophilic nature (Kobayashi et al., 2008) and have different recommended dosages (Watson, 2008). It has been suggested that the majority of side effects of statins are related to their lipophilic nature and are dose-dependent. In terms of lipophilic nature, lovastatin and simvastatin are the most lipophilic, followed by atorvastatin, fluvastatin, and pravastatin. Rosuvastatin is a relatively new statin, having a polar methane sulfonamide group, and can be placed between fluvastatin and pravastatin. While a number of studies have investigated the effects of the hydrophilic or lipophilic nature of statins on cognitive status in rodents (Vecka et al., 2004; Thelen et al., 2006; Stuart et al., 2013), similar studies in humans have been few. Therefore, we also decided to assess whether cognitive status is differently affected by their hydrophilic or lipophilic nature and/or by the dose of statins.

Four hundred and fifty-five patients taking statins were divided into three groups and analyzed according to their mini mental state examination score; group 1: 144 patients, 138 of whom taking simvastatin, and 6 lovastatin; group 2: 190 taking atorvastatin; group 3: 121 patients, 6 of whom taking fluvastatin, 89 rosuvastatin, and 26 pravastatin. Chi-square test was performed in order to establish whether lipophilic nature and/or statin dose, affect cognitive status. As shown in Table 5, no type of statin or statin dose was found to be significantly associated with mini mental state examination <24 score, indicating that statins may affect cognition irrespective of their lipophilicity and dose.

**Table 5:** **Associations between MMSE and lipophilic nature and statin dose**.

Group	MMSE < 24	MMSE ≥ 24.5	Total	χ^2^ (*P*-value)
1	94	50	144	3.666 (0.160)
2	113	77	190	
3	65	56	121	
Total	272	183	455	

**Dose**	**MMSE < 24**	**MMSE ≥ 24**	**Total**	**χ^2^ (*P*-value)**

High (40–80 mg)	32	26	58	5.845 (0.054)
Low (10–20 mg)	240	157	397	
Total	272	183	455	

MMSE, mini mental state examination

However, when different types of dementia were considered, chi-square test was found to be significant (*P* = 0.036). In particular, we found that the most lipophilic statins (group 1) were taken by 33% of Alzheimer’s disease patients, 13% of mixed dementia ones, 17% of vascular dementia ones, and 37% of mild cognitive impairment ones, while the most hydrophilic statins (group 3), by 22% of Alzheimer’s disease patients, 13% of mixed dementia ones, 38% of vascular dementia ones, and 27% of mild cognitive impairment ones, indicating that lipophilic statins more frequently give rise to the complex spectrum of non-vascular dementia, which includes mild cognitive impairment and Alzheimer’s disease, while hydrophilic statins give rise to vascular dementia.

## Discussion

Growing evidence suggests that memory impairment and age-related vision problems are closely linked in patients with dementia, therefore, vision tests have been proposed for early dementia detection (Reischies and Geiselmann, 1997). In particular, it was found that older patients with advanced macular degeneration and reduced vision may be more likely to also have cognitive impairment or problems with reasoning, learning, and memory (Clemons et al., 2006). Several possible explanations for these associations have been given: 1. macular degeneration and dementia are both chronic neurodegenerative disorders affecting an increasing number of people as they age. 2. A common characteristic of macular degeneration and cognitive impairment is nerve cell loss. 3. Retina degeneration may lead to problems with both vision and cognition. 4. It has also been hypothesized that the relationship between visual and cognitive impairment may be based on the influence of visual impairment on the level and quality of interactive experiences of older adults, suggesting that a reduced capacity to develop and maintain relationships and to participate in activities may also influence physical, mental, and psychosocial behavior (Clemons et al., 2006). In the present study, none of the cardiovascular diseases examined were associated with any type of dementia or with Alzheimer’s disease. Significant association was found between atherosclerosis and mixed dementia and vascular dementia, and also between diabetes and mixed dementia, indicating that cardiovascular diseases do not appear to be risk factors specific for dementia. By way of confirmation, a recent study pooling raw data from 10 UK general population-based prospective cohort studies – in the context of individual participant meta-analysis – concludes that there is only limited evidence that cardiovascular disease risk factors are related to death due to dementia (Batty et al., 2014).

No significant differences in mean age were observed between control groups and dementia or vision disorder groups. Mini mental state examination scores, activities of daily living ones, and instrumental activities of daily living ones indicate that patients with dementia are more depressed, have higher functional disability in basic and instrumental activities of daily living than controls. As expected, patients with poor visual acuity were more likely to be depressed and have more comorbidity than controls, both in terms of cumulative illness rating scale and severity index or comorbidity index, but, surprisingly, they have significantly lower mini mental state examination scores. When different types of vision disorder and dementia were analyzed, we found that patients diagnosed with Alzheimer’s disease had closer association with macular degeneration than patients with other forms of dementia. This study is novel in its investigation of the relationships between mild cognitive impairment, various forms of age-related dementia (Alzheimer’s disease, mixed dementia, and vascular dementia) and the four major recognized age-related vision disorders, namely: cataract, macular degeneration, glaucoma, and diabetic retinopathy. A number of studies that have evaluated the relationship between macular degeneration and cognitive impairment or dementia confirm our results. In fact, most of these studies found an association between cognitive impairment and late macular degeneration (Pham et al., 2006). Association with early macular degeneration and cognitive impairment was found only in an older population, suggesting that a pathophysiology is likely shared by macular degeneration and age-related brain diseases (Baker et al., 2009). The results of a previous study of ours were in apparent contrast: no cognitive impairment was found – as assessed by mini mental state examination – in a group of 136 macular degeneration patients admitted to the outpatient eye clinic of the University of Cagliari (Peiretti et al., 2014). However, these patients were younger and macular degeneration was not in an advanced stage. Although the results of the present study seem to support the idea that the presence of macular degeneration may be important for predicting Alzheimer’s disease, it remains to be determined whether, in the case of their concurrent presence, macular degeneration always precedes cognitive function deterioration. In this study, the chi-square test confirmed that dementia is significantly associated with vision disorders, and that Alzheimer’s disease is closely related to macular degeneration in particular.

Another purpose of this study was to explore the impact of statin and aspirin use on cognition and vision. Several studies examining the relationship between dementia and statin use suggest that taking statins lowers the risk of dementia (Jick et al., 2000). However, a recent systematic review of the literature on statins and dementia – including Alzheimer’s disease – showed that the use of statins to prevent vascular disease did not appear to prevent Alzheimer’s disease (McGuinness et al., 2009). Studies have also examined the effect of non-steroidal anti-inflammatory drugs on the development of dementia (Rist et al., 2012; Liew et al., 2013). These studies have shown that aspirin use can actually put people at increased risk of certain types of dementia (Rist et al., 2012; Liew et al., 2013). However, other studies have shown no difference in dementia risk between people who take aspirin and those who do not (Kern et al., 2012). People who were regular aspirin (Klein et al., 2012) and/or statin (Hall et al., 2001) users were also reported to be affected by vision disorders – macular degeneration in particular. In this case, too, however, conflicting results were reported.

In this study, we found that, compared to aspirin non-users, aspirin users alone were significantly positively associated with all types of dementia, taken collectively, and with mild cognitive impairment, mixed dementia, and vascular dementia in particular. Compared to statin non-users, statin users alone were significantly positively associated with all types of dementia, taken collectively and with Alzheimer’s disease. The combination of these two drugs was associated with all types of dementia, taken collectively, and with mild cognitive impairment, Alzheimer’s disease, mixed dementia, and vascular dementia in particular. The association between mild cognitive impairment, mixed dementia and vascular dementia and aspirin and statin use is not a surprise, since mild cognitive impairment, mixed dementia, and vascular dementia patients often suffer from atherosclerosis, for the treatment of which these two drugs are prescribed. On the contrary, the positive association between Alzheimer’s disease and statin use is less easy to explain, since Alzheimer’s disease patients do not seem to be associated with other age-related disorders for which statins and aspirin are prescribed.

Comparison between Control 3 and subjects with vision disorders, but without dementia, did not show significant association for statin use alone, aspirin use alone, or the combination of these two drugs, indicating that statins and aspirin do not appear to protect against age-related vision disorders.

In summary, this data, not only indicate a positive effect of statins and aspirin in lowering the risk of developing macular degeneration or Alzheimer’s disease but even seem to suggest that statins may favor Alzheimer’s disease development. In addition, it offers no evidence that statins and aspirin may be used for the improvement of vision disorders, macular degeneration in particular. The mechanism by which statins might worsen cognitive functions is unknown, but one of the prevailing hypotheses focuses on the role of cholesterol in the brain. Since cholesterol plays a fundamental role in the myelination of neurons, it has been proposed that excessive inhibition of cholesterol synthesis could lead to adverse cognitive effects (Fassbender et al., 2002). Indeed, there is some evidence that statin treatment inhibits local synthesis of cholesterol in the central nervous system (Lütjohann et al., 2004). In addition, if the effects of statins on memory and dementia are mediated directly by the central nervous system, one would predict lipophilicity to be correlated with neural effects associated with statins. Accordingly, some studies have reported that the most lipophilic statins may have a greater propensity for crossing the blood–brain barrier and affecting central nervous system activity (Serajuddin et al., 1991). Until now, only a limited number of studies have investigated cognitive problems in humans undergoing different types of statin therapy. In the present study, the analysis of the effects of statin lipophilicity on learning and memory, as determined by mini mental state examination, revealed that statins affect cognition irrespective of their lipophilicity and dose. However, when different types of dementia were analyzed, we found that lypophylic statins were more closely associated to Alzheimer’s disease and mild cognitive impairment than they were to vascular dementia.

Our results neither definitely indicate whether the regular administration of statins may induce memory changes nor whether some statins, based on their hydrophobic/lipophilic status, may be more beneficial than others: therefore, given the high number of studies showing memory loss in the population receiving statins, additional studies on statins and cognition, comparing the short- and long-term effects of various statins, are necessary.

## Conflict of Interest Statement

The authors declare that the research was conducted in the absence of any commercial or financial relationships that could be construed as a potential conflict of interest.
